# Circulating E3 ligases are novel and sensitive biomarkers for diagnosis of acute myocardial infarction

**DOI:** 10.1042/CS20140663

**Published:** 2015-03-17

**Authors:** Qiu-Yue Han, Hong-Xia Wang, Xiao-Hong Liu, Cai-Xia Guo, Qi Hua, Xiao-Hong Yu, Nan Li, Yan-Zong Yang, Jie Du, Yun-Long Xia, Hui-Hua Li

**Affiliations:** *Department of Physiology, Pathology and Pathophysiology, Beijing AnZhen Hospital the Key Laboratory of Remodeling-Related Cardiovascular Diseases, School of Basic Medical Sciences, Department of Cardiology, Beijing Chaoyang Hospital, Capital Medical University, Beijing 100069, China; †Department of Cardiology, Shanxi Province People's Hospital, Taiyuan 030012, China; ‡Department of Cardiology, Beijing Tiantan Hospital, Capital Medical University, Beijing 100050, China; §Department of Cardiology, Beijing Xuanwu Hospital Affiliated to Capital Medical University, Beijing 100053, China; ║Department of Cardiology, Institute of Cardiovascular Diseases, First Affiliated Hospital of Dalian Medical University, Dalian 116011, China

**Keywords:** acute myocardial infarction, blood, biomarkers, E3 ubiquitin ligases, Rnf207, AMI, acute myocardial infarction, CHD, coronary heart disease, CK, creatine kinase, cTns, cardiac troponins, E1, ubiquitin activating enzyme, E2, ubiquitin conjugating enzyme, E3, ubiquitin ligase, ECG, electrocardiogram, LDH, lactate dehydrogenase, ROC, receiver operating characteristic, SCD, sudden cardiac death, UPS, ubiquitin-proteasome system

## Abstract

Ubiquitin ligase (E3) is a decisive element of the ubiquitin-proteasome system (UPS), which is the main pathway for intracellular protein turnover. Recently, circulating E3 ligases have been increasingly considered as cancer biomarkers. In the present study, we aimed to determine if cardiac-specific E3 ligases in circulation can serve as novel predictors for early diagnosis of acute myocardial infarction (AMI). By screening and verifying their tissue expression patterns with microarray and real-time PCR analysis, six of 261 E3 ligases, including cardiac-specific Rnf207 and cardiac- and muscle-enriched Fbxo32/atrogin-1, Trim54/MuRF3, Trim63/MuRF1, Kbtbd10/KLHL41, Asb11 and Asb2 in mouse heart, were selected for the present study. In the AMI rats, the levels of five E3 ligases including Rnf207, Fbxo32, Trim54, Trim63 and Kbtbd10 in the plasma were significantly increased compared with control animals. Especially, the plasma levels of Rnf207 was markedly increased at 1 h, peaked at 3 h and decreased at 6–24 h after ligation. Further evaluation of E3 ligases in AMI patients confirmed that plasma Rnf207 level increased significantly compared with that in healthy people and patients without AMI, and showed a similar time course to that in AMI rats. Simultaneously, plasma level of cardiac troponin I (cTnI) was measured by ELISA assays. Finally, receiver operating characteristic (ROC) curve analysis indicated that Rnf207 showed a similar sensitivity and specificity to the classic biomarker troponin I for diagnosis of AMI. Increased cardiac-specific E3 ligase Rnf207 in plasma may be a novel and sensitive biomarkers for AMI in humans.

## Abstract 2

Because of the delayed release of troponins, early diagnosis of acute myocardial infarction (AMI) is a problem. E3 ligase Rnf207 showed higher sensitivity and specificity for diagnosis of AMI. Therefore, We concluded Rnf207 may be a novel biomarkers for AMI.

## INTRODUCTION

Acute myocardial infarction (AMI) is the leading cause of morbidity and mortality worldwide. Despite a recent substantial decline in mortality after AMI, the incidence of ischaemic heart failure in post-AMI patients is increasing [1]. Thus, an early diagnosis of AMI is required for optimizing therapy and reducing the risk of heart failure. Currently, in those patients presenting with chest pain the electrocardiogram (ECG) has limited sensitivity (50–60%) for the diagnosis of AMI. In addition, several types of biomarkers, such as cardiac troponins (cTns), myoglobin, N-terminal probrain natriuretic peptide, creatine kinases (CK) and lactate dehydrogenase (LDH) have been widely applied in clinical diagnosis of AMI patients [2–5]. Recently, miRNAs have also been recognized as novel biomarkers for early diagnosis of AMI, including miR-208α, miR-1 and miR-126 [6,7]. Among them, cardiac-specific troponin I and T, which are released as early as CK and remain elevated for as long as LDH, are currently considered as the diagnostic ‘gold standard’ for AMI. However, early diagnosis of AMI is a problem due to the delayed release of troponins, which started to be elevated within 4–6 h and peak concentrations are reached approximately 12–24 h after infarction [8–10]. Therefore, identification of novel biomarkers with high sensitivity and specificity for early stage AMI diagnosis remains to be further explored.

The ubiquitin-proteasome system (UPS) is a major pathway for the intracellular degradation of ubiquitinated or damaged proteins and involves multistep enzymatic reactions catalysed by a cascade of enzymes, including ubiquitin activating enzyme (E1), ubiquitin conjugating enzyme (E2) and ubiquitin ligase (E3). Currently, approximately 500 E3 ubiquitin ligases are identified in the human genome, which can bind to specific protein substrates and target them for degradation by the proteasome [11,12]. Many data have strongly suggested that E3 ligases play important roles in regulating diverse cellular processes, such as cell proliferation, differentiation and cell death [13]. Although the pathological functions of E3 ligases are not fully understood, some E3 ligases are reported to be expressed in a tissue-specific pattern [14]. At least 13 have been described in the heart, with 11 of these (Trim63/MuRF1, Trim54/MuRF3, Fbox32/atrogin-1, CHIP, MDM2, Nrdp1, Ozz, Nedd4-like Trim32, c-Cbl) being mechanistically characterized in muscle and cardiac atrophy, hypertrophy, metabolism, infarction and ischaemia/reperfusion injury [1,14–29]. In particular, recent studies indicate that proteasome activity or E3 ligases have been linked to the diagnosis and prognosis for cancer diseases [30]. However, little is known about whether E3 ligases can serve as early and specific biomarkers of AMI.

In the present study, we hypothesized that the cardiac-specific E3 ligases might be released into the circulating blood during AMI, and could be used to predict the myocardial damage. We measured the cardiac-specific E3 ligases in blood from rat models and patients after AMI. Our results identified circulating E3 ligase Rnf207 that can serve as a novel sensitive biomarkers for the diagnosis of AMI in patients.

## MATERIALS AND METHODS

### Animals

Age-matched male wild-type (WT) C57BL/6 mice (weighing 20±1 g) and male Sprague–Dawley rats (weighing 200±10 g) were purchased from the Animal Center, Capital Medical University. All animals were kept 1 week at 22°C and 55% relative humidity in a 12-h day/night lighting environment with free access to food and water. All procedures were approved by the Animal Care and Use Committee of Capital Medical University and the experiments complied with the Guide for the Care and use of Laboratory Animals published by the US National Institutes of Health (NIH Publication No. 85–23, revised 1996).

### AMI rat model

AMI was induced by left coronary artery ligation as described [31,32]. For details, see Supplementary material online.

### Microarray assays

To identify cardiac-specific E3 ligases, we first performed gene global expression profiling in mouse heart, kidney, brain and aorta (*n*=2–3 per group) using Affymetrix GeneChip mouse Genome 230 2.0 array according to the manufacturer's instructions (Affymetrix) [24,33]. On the GeneChip Mouse Genome 430 2.0 Array, over 45,000 probe sets analyse the expression level of over 39000 transcripts and variants from over 34000 well characterized mouse genes. The microarray data in the present study have been deposited in NCBI's Gene Expression Omnibus.

### Quantitative real-time PCR analysis

Total RNA was extracted with TRIzol (Invitrogen) from mouse tissues of heart, skeletal muscle, liver, spleen, lung, kidney, brain and aorta according to manufacturer's instructions (Invitrogen). The gene expression levels were quantified relative to the expression of GAPDH. The primer sequences are shown in Table S1 (see Supplementary material online). For details, see Supplementary material online.

### Population

We analysed 65 AMI and 31 non-AMI patients with chest distress and pain admitted to the Department of Cardiology in Beijing Xuanwu Hospital, Shanxi Province People's Hospital and First Affiliated Hospital of Dalian Medical University between September 2013 and April 2014. These patients with AMI were clinically diagnosed based on a combination of several criteria, and the criteria see Supplementary material online.

### Plasma preparation

Blood samples for ELISA detection were obtained from AMI, non-AMI patients and healthy control and were processed within 1.0 h of collection by centrifugation. The supernatants were transferred to from each tube to fresh 1.5 ml tubes and stored at −80°C for future analysis.

### Measurement of E3 ligases

The plasma levels of E3 ligases and cardiac troponin I (cTnI) were measured with an ELISA according to the manufacturer's instructions (CUSABIO). For details, see Supplementary material online.

### Statistical analysis

All values are presented as mean+S.D. unless otherwise indicated. As the data that fit the homogeneity of variance, one-way ANOVA was applied for multiple comparisons. The receiver operating characteristic (ROC) curves were established for discriminating AMI patients from healthy adults. All statistical calculations were performed by the SPSS 17.0. *P*<0.05 was considered statistically significant.

## RESULTS

### Identification of cardiac-specific E3 ligases

**Figure 1:**
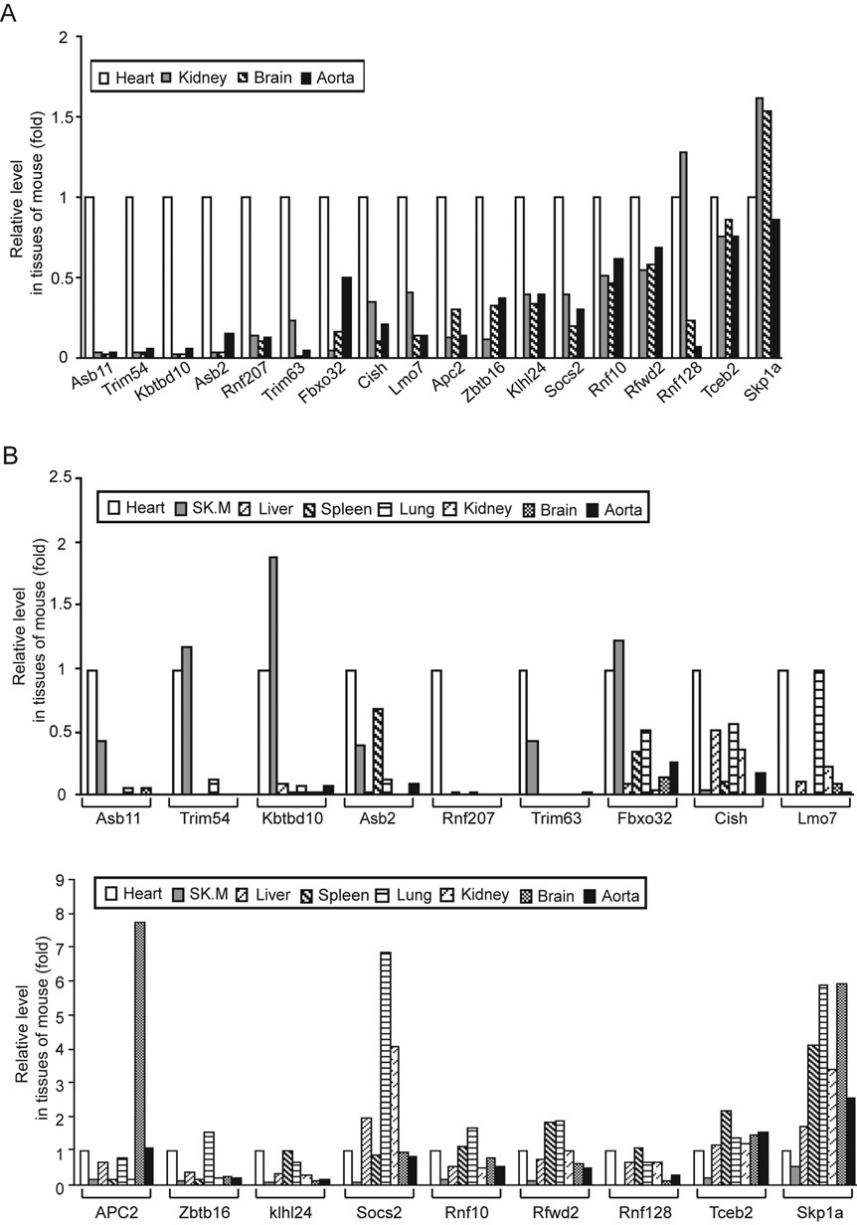
Detection of E3 ligases specifically expressed in the mouse heart (**A**) Microarrays analysis of ubiquitin E3 ligases expression in heart, aorta, kidney and brains from mouse. (**B**) Real-time PCR analysis of ubiquitin E3 ligases expression in eight organs from mouse (*n*=9 per group). Sk.M, skeletal muscle.

In our preliminary experiments, we first determined which E3 ligases were specifically or highly expressed in the mouse hearts compared with other tissues. The mRNA microarray assays were performed in the heart, kidney, brain and aorta from WT mice. A total 34000 genes were screened, and 261 E3 ligases were detected in four tissues (see Supplementary material online, Table S2). Among them, sixteen E3 ligases that are highly expressed in the heart but lower in the other three tissues, and two E3 ligases that are ubiquitously expressed in four tissues as controls were selected (Table S2, Figure 1A).

To confirm the expression patterns of those E3 ligases in the heart, kidney, brain and aorta obtained from microarray, qPCR analysis was performed. Scatter plot analysis of gene expression values showed a good correlation between microarray and qPCR (see Supplementary material online, Figure S1). To further determine which E3 ligases are highly and specifically expressed in the heart, we also detected the expression of those E3 ligases in other major organs, including skeletal muscle, liver, spleen and lung. qPCR analysis revealed that Rnf207 was greatly and specifically expressed in the heart. Asb11, Trim54, Kbtbd10, Trim63 and Fbxo32 were predominantly expressed in the heart and skeletal muscle, some of them are in agreement with previous reports [27,34–36]. Others including Asb2, Cish, Lmo7, Apc and so on were expressed in different tissues (Figure 1B). We therefore chose Rnf207, Fbxo32, Trim54, Trim63, Kbtbd10 and Asb11 as candidate biomarkers of AMI.

### Cardiac-specific E3 ligases are increased in blood from AMI rats

To investigate whether cardiac E3 ligases can be released into blood and be detected with their increase in circulating blood after AMI, we generated an AMI rat model by left coronary artery ligation. Rat cardiac severity and function were evaluated with TTC staining and echocardiography at 24 h of ligation, respectively. We found that the infarct size was about 33±4% of left ventricular area. Although MI can slightly reduce cardiac function, there were no statistically significant differences (Supplementary Figure 2).

**Figure 2:**
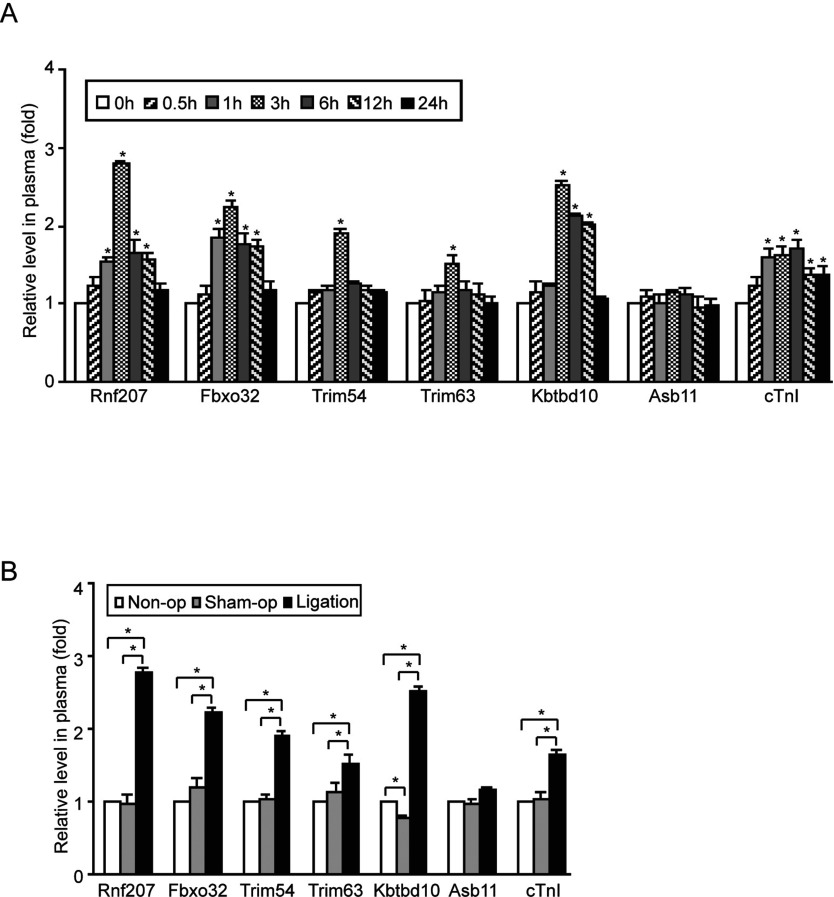
Measurement of plasma E3 ligases in rats with acute myocardial infarction (**A**) The plasma was collected from the rats at different times (0, 0.5, 1, 3, 6, 12 and 24 h) after coronary artery occlusion (*n*=8–10 per group), and the levels of circulating E3 ligases and cTnI were measured by using ELISA kit. (**B**) The plasma was collected from rats of non-operation (non-op), sham-operation (sham-op) and ligation groups at 3 h after coronary artery occlusion (*n*=5 per group). Data are expressed as mean±S.D. *, *P*<0.05.

Blood samples were collected from the rats at different time points (0.5, 1, 3, 6, 12 and 24 h) after permanent ligation, and the levels of circulating troponin I (cTnI) and E3 ligases were measured by using ELISA kits. We found that plasma cTnI was markedly increased (1.6-fold) at 1 h and maintained a high level until 24 h (Figure 2A). Notably, the plasma levels of Rnf207 and Fbxo32 were also markedly increased (1.6- or 1.9-fold, respectively) at 1 h, peaked (2.8- or 2.2-fold, respectively) at 3 h and decreased at 6–24 h after ligation, whereas the plasma levels of Trim54, Trim63, and Kbtbd10 did not increase at 1 h, but significantly increased at 3 h, then decreased to baseline at 6–24 h (Figure 2A). The levels of Asb11 did not change considerably during the AMI procedure. Interestingly, the levels of E3 ligases and cTnI did not have an obvious increase at 0.5 h after ligation (Figure 2A).

### Plasma levels of E3 ligases in rat with thoracotomy

Since Trim54, Trim63, Fbxo32, Kbtbd10 and Asb11 (as a control) are also highly expressed in skeletal muscle, we then assessed whether muscle injury, which is due to surgical procedure of thoracotomy used in the present study, had any effect on plasma levels of these E3 ligases. We generated three experimental groups: rats received anaesthetic agent only (control group), rats received thoracotomy only (sham group), and rats received thoracotomy followed by ligation (ligation group), and collected blood samples from rats at 3 h after ligation. As shown in Figure 2B, the plasma levels of Rnf207, Fbxo32, Trim54, Trim63 and Kbtbd10 in the ligation group were significantly higher than in the control (non-operation) or sham (sham-operation). Moreover, the change of cTnI level was similar with these E3 ligases. There was no difference between control and sham groups. In contrast, Asb11 plasma levels did not change. Thus, thoracotomy, unlike MI, did not increase the plasma levels of the E3 ligases under study.

### Plasma levels of cardiac-specific E3 ligases are increased in patients after AMI

**Figure 3:**
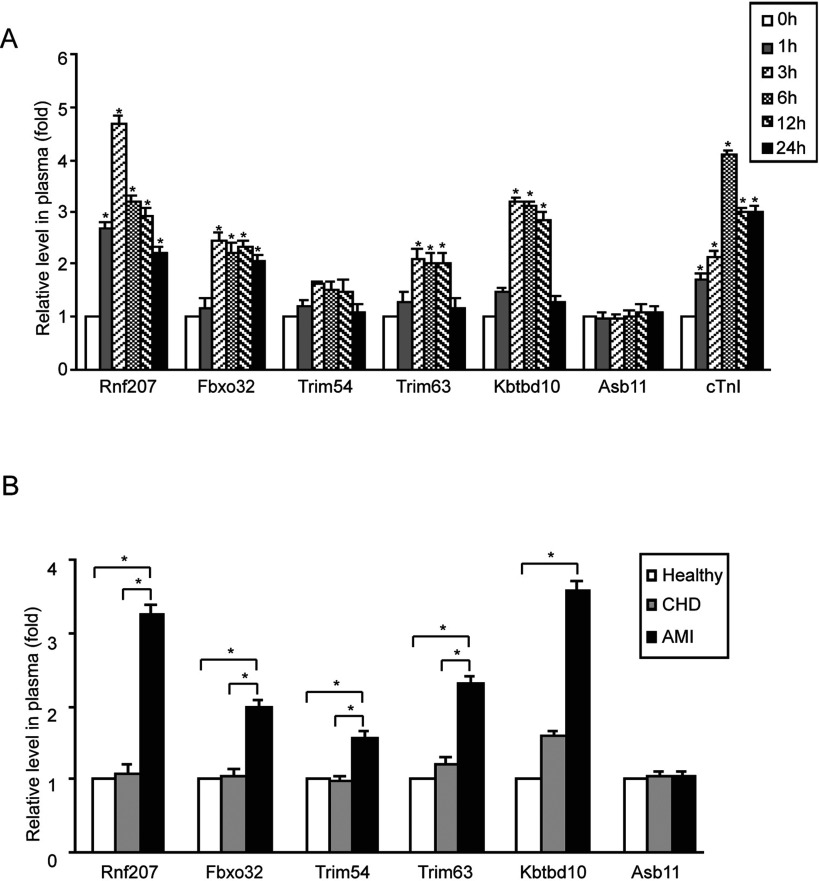
Measurement of plasma E3 ligases in patients with acute myocardial infarction (**A**) The plasma was collected from patients at various time points (0.5, 1, 3, 6, 12 and 24 h) and the levels of circulating E3 ligases and cTnI were measured by using ELISA kit (*n*=12–15 per group). (**B**) The plasma was collected from acute myocardial infarction (*n*=65), patients with coronary heart disease but without acute myocardial infarction (coronary heart disease, *n*=31), and healthy volunteers (healthy, *n*=28). Data are expressed as mean±S.D. *, *P*<0.05.

To confirm whether increased E3 ligases in circulating blood observed in a rat model of AMI can be reproduced in human patients with AMI, we collected blood samples from patients at various time points (1, 3, 6, 12, 24, 48 and 72 h) after AMI. The clinical characteristics of all the patients are summarized in Table 1. There were no significant differences in age, sex, history of hypertension, hyperlipidaemia and smoking status among the three groups. There was also no significant difference in plasma level of E3 ligases between female and male persons, neither in the control group nor in AMI patients (data not shown). Consistent with findings from AMI rats (Figure 2A), the plasma level of Rnf207 was significantly increased at as early as 1 h, peaked at 3 h, and then began to decrease at 6–24 h after AMI (Figure 3A). The plasma levels of Fbxo32, Trim54, Trim63 and Kbtbd10 were also observed to increase in patients with AMI times, though to a lesser extent, after AMI (Figure 3A). After 24 h, the levels of Rnf207 and Fbxo32 in plasma were decreased, and other E3 ligases did not change significantly (Supplementary Figure 3). However, the level of Asb11 did not change considerably during AMI (Figure 3A).

**Table 1:** Clinical characteristics of patients with and without acute myocardial infarction

Characteristics	Healthy adults (*n*=28)	Total patients (*n*=96)	AMI (*n*=65)	CHD (*n*=31)	P1	P2
Age (years)	64.67±11.56	64.26±12.24	64.3±13.5	64.2+9.03	0.604	0.818
Male/female (*n*/*n*)	17/11	72/24	50/15	22/9	0.755	0.853
Current smoking, *n* (%)	6 (21.4)	55 (57.3)	37 (56.9)	18 (58.1)	0.855	0.843
Hypertension, *n* (%)	14 (50.0)	61 (63.5)	42 (64.6)	19 (61.3)	0.602	0.714
Hyperlipidaemia, *n* (%)	2 (7.1)	3 (3.1)	2 (3.1)	1 (3.2)	0.503	0.544
Fasting glucose (mmol/l)	5.64±1.26	6.21±3.32	6.28±3.78	6.14+3.19	0.669	0.051
DM, *n* (%)	3 (10.7)	35 (36.5)	27 (41.5)	8 (25.8)	0.125	0.011
SBP (mmHg)	126.71±6.18	128.23±12.6	124.7±5.37	131.73±10.8	0.607	0.673
DBP (mmHg)	76.18±4.12	79.16±6.54	73.6±3.39	84.71±4.59	0.358	0.364
TC (mmol/l)	5.04±1.71	5.91±3.12	5.85±1.32	4.61±0.99	0.923	0.425
TG (mmol/l)	2.78±0.86	2.41±1.03	2.74±1.33	2.34±1.16	0.726	0.275
HDL (mmol/l)	1.32±0.76	1.65±0.35	1.28±0.07	1.31±0.92	0.245	0.679
LDL (mmol/l)	3.34±0.43	2.86±0.29	2.6±0.22	3.12±0.32	0.114	0.365
WBC (10^9^/l)	5.59±1.42	6.21±1.51	6.31±1.53	6.11±1.5	0.965	0.678
Cr (mmol/l)	75.17±18.4	72.19±19.18	73.12±17.18	71.26±16.89	0.722	0.186

DM, diabetes mellitus; SBP, systolic blood pressure; DBP, diastolic blood pressure; TC, total cholesterol; TG, total glyceride; HDL, high-density lipoprotein; LDL, low-density lipoprotein; WBC, white blood cell; Cr, creatinine. P1: comparison between patients with AMI and CHD. P2: comparison among patients with AMI, with CHD, and healthy.

We next investigated whether the plasma levels of E3 ligases in AMI patients actually can reflect the onset of AMI. We examined the plasma Rnf207, Fbxo32, Trim54, Trim63, Kbtbd10 and Asb11 in three groups (the AMI, coronary heart disease (CHD) and healthy groups). As shown in Figure 3B, plasma levels of Rnf207, Fbxo32, Trim54, Trim63 and Kbtbd10 in the AMI group were significantly higher than that in other groups. However, there were no statistically significant differences in the levels of those E3 ligases between the healthy and CHD groups.

### Evaluation of plasma E3 ligases as novel biomarkers for AMI

**Figure 4:**
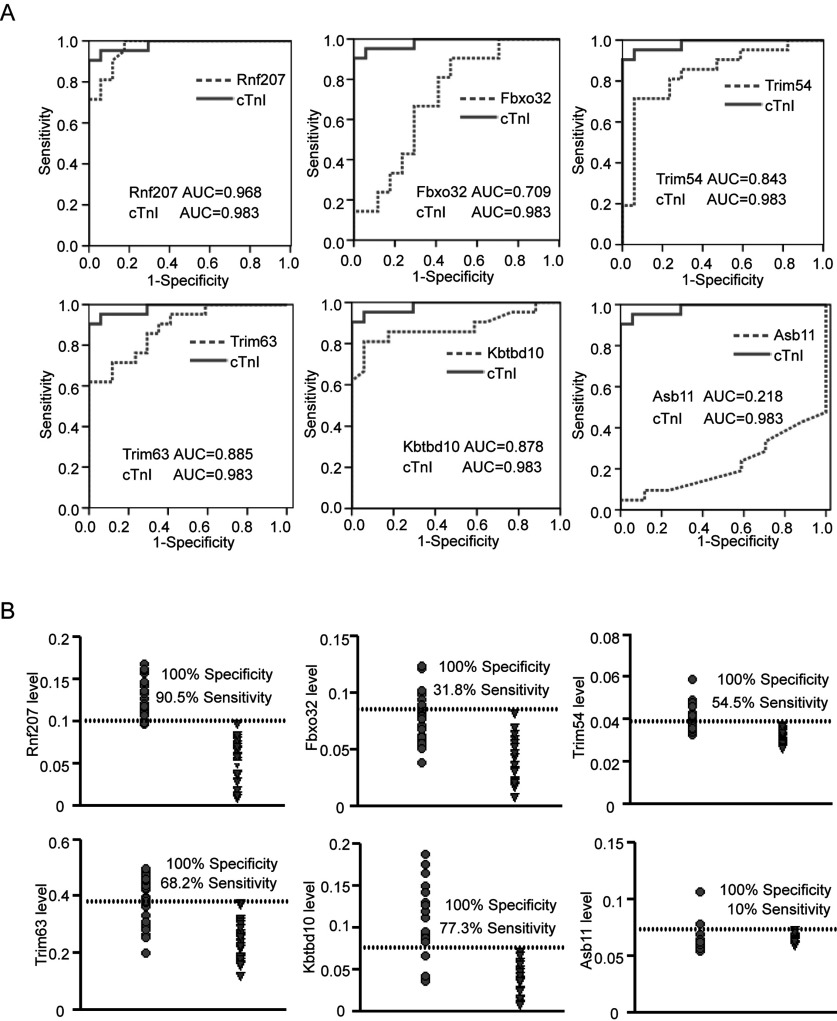
Evaluation of plasma E3 ligases for the diagnosis of acute myocardial infarction (**A**) Receiver operating characteristic curves (ROC) were drawn of 65 AMI patients and 28 healthy volunteers with the data of plasma ubiquitin E3 ligases. AUC, the area under curve. (**B**) Sensitivity and specificity of plasma ubiquitin E3 ligases levels in the diagnosis of AMI. The dashed line indicates a 100% specificity threshold. Filled circle, AMI patients; inverted triangle, healthy volunteers.

To explore whether elevated E3 ligases operate as potential biomarkers for diagnosis of AMI, the receiver operating characteristic (ROC) analysis was established for discriminating AMI patients (*n*=65) from healthy groups (*n*=28). Simultaneously, plasma cTnI level was measured by ELISA assays. The ROC curves of Rnf207, Fbxo32, Trim54, Trim63 and Kbtbd10 reflected strong separation between AMI and non-AMI groups, with an area under curve (AUC) of 0.968, 0.709, 0.843, 0.885 and 0.878 respectively, compared with initial cTnI with an AUC of 0.983. In contrast, AUC of Asb11 was lower (0.218) (Figure 4A). Furthermore, plasma level of Rnf207 could detect individuals with AMI with 90.5% sensitivity at 100% specificity, whereas the plasma levels of Fbxo32, Trim54, Trim63 Kbtbd10 and Asb11 were less sensitive (31.8%, 54.5%, 68.2%, 77.3% and 10%, respectively) for diagnosis of AMI (Figure 4B). Collectively, these results demonstrate that among the 6 E3 ligases investigated, circulating Rnf207 displayed the highest sensitivity and specificity for early diagnosis of AMI.

## DISCUSSION

In the present study, we have identified E3 ligases that can be clinically practicable biomarkers for AMI diagnosis. In rat AMI model, we found that the plasma levels of E3 ligases were markedly increased after AMI, especially the cardiac-specific Rnf207, which becomes elevated as early as 1 h after AMI, suggesting that cardiac E3 ligases could be released into circulating blood after heart damage occurred. We further confirmed that the plasma Rnf207 was markedly increased in plasma from AMI patients, indicating for the first time that monitoring the plasma levels of Rnf207 could be applied in clinical diagnosis of AMI.

The UPS plays a fundamental role in the development of the cardiovascular diseases. E3 ligases are key enzymes of UPS that are expressed in the different tissues to regulate protein degradation. Numerous studies have demonstrated that several E3 ligases are highly expressed in the heart and skeletal muscle, and play critical roles in regulating cardiac hypertrophy and apoptosis in response to various stresses [27,35,36]. For example, Fbxo32, as an F-box protein containing an E3 ligase, regulates cardiac hypertrophy, ischaemic injury and cardiomyocyte apoptosis through degradation of calcineurin and MKP-1 degradation [14,19]. Trim63 inhibits cardiac hypertrophy and protects against cardiac infarction through degradation of tropnin I, PKCε and c-Jun [37,38]. Trim54 is essential for maintenance of ventricular integrity and function after myocardial infarction [17]. Rnf207 is an ubiquitin ligase that is specifically expressed in heart. It is reported that genetic variation in RNF207 may influence the duration of QT interval that could predisposes to ventricular arrhythmias and sudden cardiac death (SCD) [39,40]. Importantly, the expression of several E3 ligases such as Fbxo32 and TRIM63 are induced by ischaemia injury [14,38]. In the present study, our data for the first time showed that Rnf207 was highly expressed only in the mouse heart. Others including Trim63, Trim54, Fbxo32 and Asb11 were highly expressed both in the mouse heart and skeletal muscle (Figure 1). In parallel, the plasma levels of these E3 ligases particularly Rnf207 were significantly increased within 1–3 h of myocardial injury in rats and patients (Figures 2 and 3). However, skeletal muscle injury by thoracotomy did not affect the plasma levels of the E3 ligases (Figure 2B). Thus, our results clearly support the hypothesis that E3 ligases, which are highly expressed in the heart, may leak out of damaged cells into the circulating blood earlier than cTnI, and thereby serve as biomarkers for identifying AMI.

A rapid diagnosis of AMI is critical for appropriate management of patients with chest pain. The increased serum troponins can detect extremely small amounts of myocardial necrosis (<1.0 g). Previous studies showed that the plasma cTnI level was increased at 1.5–4 h, peaked at 4–8 h [7,41], whereas an other study indicated that serum cardiac troponin I was increased at 4–6 h and reached a mean peak level at 18 h [8]. Thus, identification of novel biomarkers is important to establish an early diagnosis of AMI. As several studies have shown that some E3 ligases, including Hdm2 and the F-box protein Skp2, are highly expressed in a number of human cancers, and also present in serum, it is possible that these E3 ligases can be further developed as useful cancer biomarkers [30]. Recently, several E3 ligases such as Fbxo32, Trim63, Trim54 and CHIP have been implicated in the development of AMI [14,17,28,29], but no E3 ligases have been established as biomarkers of AMI. In the present study, we found that plasma cTnI level was increased at 1 h and reached a peak at 6 h in AMI patients, which was similar to previous results [7,41]. Furthermore, our data showed that the level of Rnf207 in plasma from rats and patients was significantly increased as early as 1 h and peaked at 3 h after AMI. Notably, Rnf207 was markedly increased by 4.7-fold in AMI patients compared with that in non-AMI patients within 3 h of the onset of symptoms, when the cTnI level was still at normal level in AMI patients (Figure 3A). Finally, by receiver operating characteristic curve analysis, among the six E3 ligases analysed, Rnf207 exhibited the highest sensitivity and specificity for diagnosing AMI (Figure 4). Thus, plasma Rnf207 is a more reliable biomarker, and may be superior to cTnI for detecting early (within 3 h) myocardial injury in individuals after AMI.

In conclusion, the present study gives the first report on the diagnostic value of circulating E3 ligases in AMI. Our data support that circulating Rnf207 may be a novel predictor, which appears to reflect early AMI injury, thus providing a sensitive biomarker for early monitoring and evaluation of AMI. However, research limitations included that the sample size is small. Therefore, larger multicentre studies are warranted to evaluate the clinical significance of circulating E3 ligases as a new biomarker of AMI in humans.

## CLINICAL PERSPECTIVES

• Because of the delayed release of troponins, early diagnosis of AMI is a problem. Therefore, identification of novel biomarkers with high sensitivity and specificity for early stage of AMI diagnosis remains to be further explored.
• Rnf207 showed a similar sensitivity and specificity to the classic biomarker troponin I for diagnosis of AMI.
• Increased cardiac-specific E3 ligase Rnf207 in plasma may be a novel and sensitive biomarkers for AMI in humans.

## Online data

Supplementary data
