# Supplementary material for: Circulating E3 ligases are novel and sensitive biomarkers for diagnosis of acute myocardial infarction
Source: Clin Sci (Lond). 2015 Mar 17;128(Pt 11):751–60. doi: 10.1042/CS20140663 (PMC4557400; doi:10.1042/CS20140663)
Supplement: Supplementary data [file cs1280751ntsadd.pdf]

SUPPLEMENTAL MATERIAL

METHODS

Animals and treatment

Sprague-Dawley rats (n=8-10 per group) were anesthetized with isoflurane (induction: 3% isoflurane, maintenance: 1.5-2.0%, 1L/min oxygen level, Abbott laboratories, Chicago, USA). The left thoracotomy was performed to expose the hearts. Myocardial ischemia was induced by ligating the left anterior descending coronary artery (LAD) using a 3–0 silk suture, and a small vinyl tube was placed on top of the vessel to form a snare for permanent coronary occlussion. After myocardial infarction 24 h, cardiac function was evaluated by echocardiography. We then performed 2, 3, 5-triphenyl-2H-tetrazolium chloride (TTC) staining. Briefly, after MI, the heart was quickly excised and frozen for 1 hour at -20□. Each heart was then sectioned into 2.0 mm portions, and then incubated at 37□ in 1% TTC-PBS for 10 min. The infarct area consists of the TTC-negative staining region. Post-procedural analgesia was applied by subcutaneous (s.c.) injection of buprenophine 0.2 mg/100 g /12h for up to 24 h (if applicable). Blood samples were collected before the operation (0 h) and at 1, 3, 6, 12, and 24 h after the ligation under general anaesthesia (i.p. injection of 100 mg/kg 1% pentobarbital sodium. Sigma-Aldrich). Mice used to isolating of the different tissues for the micro-array and qPCR analysis were sacrificed by cervical dislocation.

Quantitative real-time polymerase chain reaction analysis

Total RNAs of mouse tissues was extracted with TRIzol (Invitrogen). The first-strand cDNA was synthesized with moloney murine leukemia virus reverse transcriptase (Promega, Southampton, UK). The mRNA levels were analyzed by using TaqMan miRNA quantitative real-time polymerase chain reaction (qPCR) assay according to the protocol of the manufacturer (Applied BioSystems, Inc.) as described [1-3].

Population

These patients with AMI were clinically diagnosed based on combination of several criteria: (1) ischemic symptoms; (2) biochemical markers (cardiac troponin I >0.1 ng/mL); (3) pathological Q wave; (4) coronary angiography [4, 5]. Among them, 82% patients had left coronary artery occlusion; 18% patients had right coronary artery occlusion. According to the cardiac function of Killip grade in patients, 45% were grade I, 51% were grade II, and others were grade III or IV. After diagnosed of myocardial infarction, the patients were given routine treatment, such as anti-platelet drugs, anti-anginal drugs, *et al*. The blood samples of patients with AMI were acquired at 1 h (± 30min), 3 h (± 30min), 6 h (± 30min), 12 h (± 30min), 24 h (± 30min) , 48 h (± 30min), and 72 h (± 30min) after the onset of ischemic symptoms including retrosternal pain, fever, gastrointestinal symptoms, *et al*. The non-AMI patients with distress and chest pain were separated by the outcomes of coronary angiography, including 31 patients with coronary heart disease (CHD). In addition, 28 healthy adult volunteers (17 men, 11women; normal ECG and no history of cardiovascular diseases) were enrolled in this study. Clinical characteristics of patients with and without acute myocardial infarction and healthy controls were presented in Table

1. The protocol of this study was performed according to the principles of the Declaration of Helsinki, and approved by the Medical Ethics Committee in Beijing Xuanwu Hospital, Shanxi Province People's Hospital and First Affiliated Hospital of Dalian Medical University, written informed consent was obtained from each patient.

Measurement of E3 ligases

The plasma levels of E3 ligases and cardiac troponin I (cTnI) were measured with an enzyme-linked immunosorbent assay (ELISA) according to the manufacturer's instructions (CUSABIO, America). Briefly, serum samples (100 μl per well) were incubated for 2 hour at 37℃. Standard curves for quantification were established, using purified E3 ligases in a concentration range from 0 to 10 ng/mL. The biotin-labeled monoclonal anti-E3 ligase antibody was used for detection in a 1:100 dilution and incubated for 1 hour at 37℃. Bound antibodies were detected after labeling with HRP-avidin. Microtiter plates were read at 450 nm after 30 minutes of incubation with 3, 3', 5, 5-tetramethylbenzidine (TMB). Intra-assay and interassay variations detected in serum samples of known high or low E3 ligase concentrations were lower than 10%. The recovery of E3 ligase was between 95% and 105% when purified human immunoglobulins were proteasome concentrations, indicating that serum E3 ligase detections in patients with MM are not influenced by serum immunoglobulin levels.

REFERENCES

1 Yang, K., Zhang, T.P., Tian, C., Jia, L.X., Du, J., Li, H.H. (2012) Carboxyl terminus of heat shock protein 70-interacting protein inhibits angiotensin II-induced cardiac remodeling. Am.J. Hypertens. **25**,994-1001

2 Jiang, H.M., Wang, H.X., Yang, H., Zeng, X.J., Tang, C.S., Du, J., Li, H.H. (2013) Role for granulocyte colony stimulating factor in angiotensin II-induced neutrophil recruitment and cardiac fibrosis in mice. Am. J. Hypertens. **26**,1224-1233

3 Wang, H.X., Yang, H., Han, Q.Y., Li, N., Jiang, X., Tian, C., Du,J., Li, H.H. (2013) NADPH oxidases mediate a cellular "memory" of angiotensin II stress in hypertensive cardiac hypertrophy. Free Radic. Biol. Med. **65**,897-907

4 Thygesen, K., Alpert, J.S., White, H.D. (2007) Universal definition of myocardial infarction. J. Am. Coll. Cardiol. **50**,2173-2195

5 Morrow, D.A., Cannon, C.P., Jesse, R.L., Newby, L.K., Ravkilde, J., Storrow, A.B. (2007) National Academy of Clinical Biochemistry Laboratory Medicine Practice Guidelines: Clinical characteristics and utilization of biochemical markers in acute coronary syndromes. Circulation **115**,e356-375

Figure legends:

Figure S1 Scatter plot of relative changes in gene expression of four organs as determined by microarray analysis and qPCR assay. Ten genes of each organ were randomly selected.

Figure S2 Evaluation of cardiac function in rat. After 24 h of ligation, echocardiography was performed on rats. Data are expressed as mean ± standard deviation (n= 8 per group). *, $P < 0.05$.

Figure S3 Measurement of plasma E3 ligases in patients with acute myocardial infarction. The plasma was collected from patients at 24, 48, and 72 h and the levels of circulating E3 ligases were measured by using ELISA kit (n=8-15 per group). Data are expressed as mean ± standard deviation. *, $P < 0.05$.

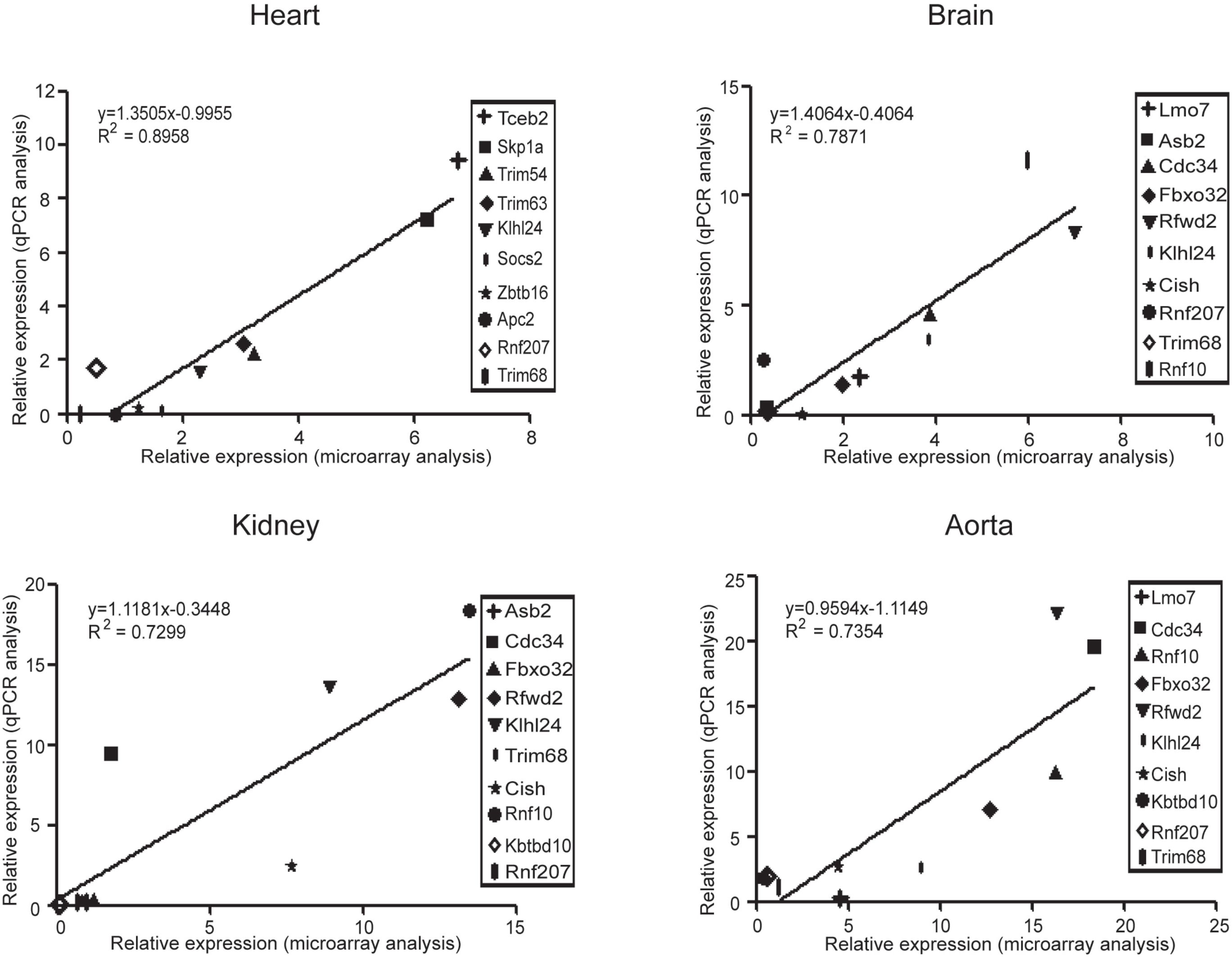

Supplmental Figure S1

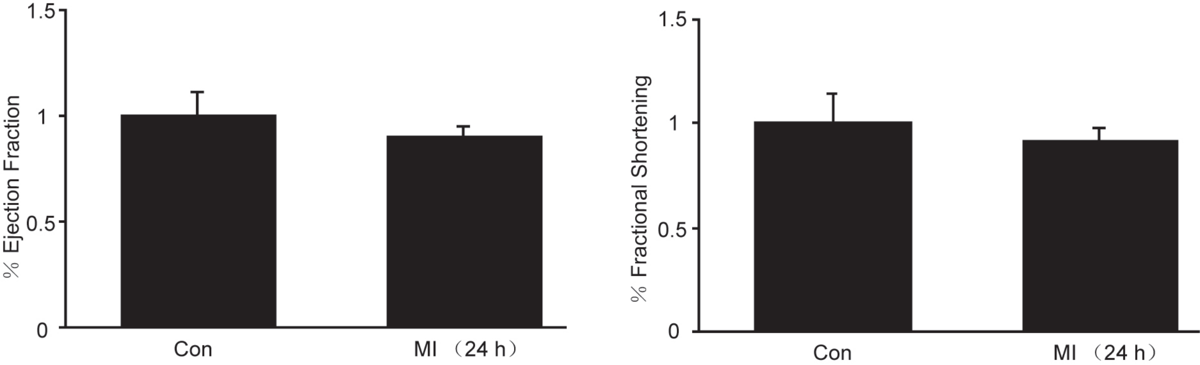

Supplemental Figure S2

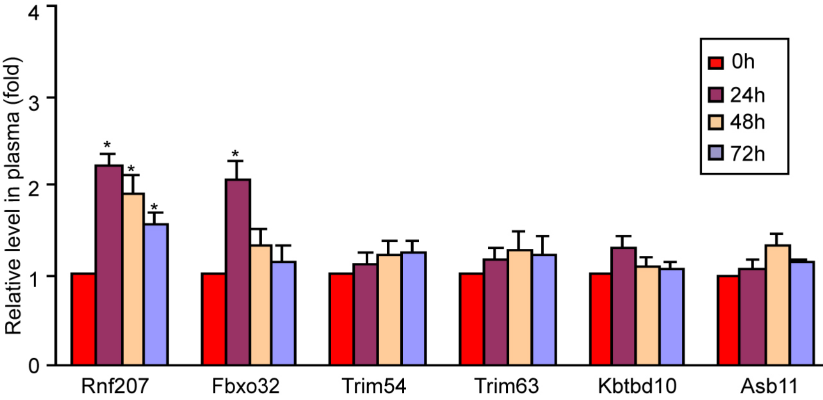

Supplemental Figure S3

Supplemental Table s1. Primer sequences used in the quantitative real-time PCR analysis

Gene symbol	Forward primer	Reverse primer
Kbtbd10	AGGATCAACCCCTGCAATCG	TTGCAGCCACAGGATCGTAG
APC2	ACTGGACCAGGAGAGATGCT	CTGCTGCCGAATCAGATCCA
Trim63	GGCGAGACAGTCGCATTTCA	ATTCGCAGCCTGGAAGATGT
Fbxo32	TCAAAGGCCTCACGATCACC	TCAAACGCTTGCGAATCTGC
Cish	TCGGGAATCTGGGTGGTACT	GGGTGCTGTCTCGAACTAGG
Zbtb16	TGAACTCTGCGGAAAACGGT	GCCATGTCCGTGCCAGTAT
Klhl24	CCGGCAGGAAAACTGTGGTA	ATTGCAGCCACTCCTGTGAT
Socs2	CGCAGAAAAACTCGGTTGCT	TCACCCACAGATCGCCTCC
Lmo7	GCCCAGATCCTACACGATGG	CACTGGCTGAGCCCGAAATA
Rnf10	TCTACCAGGCGGAAGATGGA	TACCTATGACGCTGCCGAAC
Rfwd2	GCACTATCAGACGGGGAGTC	CAGCTGCAGATGGCTTTTGG
Rnf128	CTAAGCCTTGGATTGCGCTG	CCCCAGCACCAAAATTTGCC
Skp1a	GGAGCACCATGCCTACGATA	GGTGCACCACTGAATGACCT
Tceb2	CACCGTGTTCGAACTGAAGC	AGGTGTCATCTGCTCGGAAG
Rnf207	AGCAATTTTTGCGCCTCTGG	ACTAGGGTCTGGTGCTGGC
Trim54	AGAGCGCCGCGGGATG	AGAGGATTAGAGGCCTGGAAGA
Asb11	GCCTTGCTAGAAAACGGTGC	TCTGTGACCTCGCTTCACTG
Asb2	TTCTGTGAGTTCCTGTCGGC	GTCTCGGAGGTTCTGCCTTC
GAPDH	TCCCAGAGCTGAACGGGAAG	TCAGTGGGCCCTCAGATGC

Supplemental Table s2. The mRNA levels of E3 ubiquitn ligases in mouse tissues detected by microarray assay

Gene ID	Gene Title	Gene Symbol	Geom mean of intensities			
			Heart	Kidney	Brain	Aorta
1417499_at	autocrine motility factor receptor	Amfr	1106.417	1737.039	1501.594	1110.215
1460395_at	anaphase promoting complex subunit 10	Anapc10	340.146	243.481	306.501	218.495
1433615_at	Anaphase promoting complex subunit 11	Anapc11	396.514	243.432	385.729	209.756
1430138_at	anaphase promoting complex subunit 13	Anapc13	1998.456	1369.963	776.655	1554.395
1416383_a_at	anaphase promoting complex subunit 2	Anapc2	260.450	326.558	425.554	264.180
1455534_s_at	anaphase promoting complex subunit 4	Anapc4	322.745	222.781	337.303	217.722
1415806_at	anaphase-promoting complex subunit 5	Anapc5	2312.431	1693.747	1969.938	2271.417
1426465_at	anaphase promoting complex subunit 7	Anapc7	425.853	434.318	353.224	476.254
1417516_at	ankyrin repeat and IBR domain containing 1	Ankib1	70.450	529.701	217.178	149.878
**1438559_x_at**	**adenomatosis polyposis coli**	**Apc2**	**418.261**	**49.247**	**126.028**	**54.345**
1443814_x_at	ariadne ubiquitin-conjugating enzyme E2 binding protein homolog 1 (Drosophila)	Arih1	366.146	346.935	439.959	337.046
1428339_at	ariadne homolog 2 (Drosophila)	Arih2	1760.542	1232.894	1616.798	1459.253
1429137_at	ankyrin repeat and SOCS box-containing 1	Asb1	97.588	82.058	110.489	49.129
**1447982_at**	**ankyrin repeat and SOCS box-containing 11**	**Asb11**	**3169.633**	**92.190**	**52.832**	**82.435**
**1423732_at**	**ankyrin repeat and SOCS box-containing 2**	**Asb2**	**1506.398**	**47.506**	**36.034**	**224.075**
1415790_at	ankyrin repeat and SOCS box-containing 3	Asb3	79.867	83.143	126.538	69.585
1438315_x_at	alpha thalassemia/mental retardation syndrome X-linked homolog (human)	Atrx	527.461	423.845	1060.063	510.576
1437993_x_at	BCL6 interacting corepressor	Bcor	64.966	46.716	62.991	65.946
1422820_at	bifunctional apoptosis regulator	Bfar	1595.497	589.396	661.887	1135.562
1428211_at	baculoviral IAP repeat-containing 2	Birc2	244.059	176.736	292.603	199.503
1418125_at	baculoviral IAP repeat-containing 3	Birc3	229.658	210.901	91.259	212.171
1424857_a_at	Bmi1 polycomb ring finger oncogene	Bmi1	483.448	451.640	350.534	396.022
1455316_x_at	BRCA1 associated protein	Brap	179.961	433.670	247.663	363.095
1416210_at	BRCA1/BRCA2-containing complex, subunit 3	Brcc3	833.989	452.935	407.353	301.203
1460276_a_at	beta-transducin repeat containing protein	Btrc	68.552	56.409	386.827	27.358
1435475_at	Casitas B-lineage lymphoma (Cbl), mRNA	Cbl	188.621	134.820	13.944	195.262
1423351_at	Casitas B-lineage lymphoma b	Cblb	70.220	92.067	253.276	119.995
1435967_s_at	Casitas B-lineage lymphoma-like 1	Cbll1	159.657	196.757	281.263	111.902
1435017_at	CDC16 cell division cycle 16 homolog (S. cerevisiae)	Cdc16	446.180	456.999	10.917	409.964
1449706_s_at	cell division cycle 20 homolog (S. cerevisiae)	Cdc20	57.554	54.134	52.678	40.332
1451825_a_at	CDC23 (cell division cycle 23, yeast, homolog)	Cdc23	86.464	63.745	115.029	85.000
1436860_at	cell division cycle 26	Cdc26	428.043	317.263	31.851	290.771
1449118_at	cell division cycle 27 homolog (S. cerevisiae)	Cdc27	139.866	170.212	12.361	14.033
1422684_a_at	cell division cycle 34 homolog	Cdc34	1489.266	89.422	386.904	917.267
1419743_s_at	cell growth regulator with ring finger domain 1	Cgrrf1	697.537	826.741	682.061	1135.562
1418816_at	checkpoint with forkhead and ring finger domains	Chfr	162.143	161.639	184.867	184.091

**1455958_s_at**	**cytokine inducible SH2-containing protein**	**Cish**	**1101.435**	**383.994**	**113.143**	**222.115**
1423262_a_at	CCR4-NOT transcription complex, subunit 4	Cnot4	384.019	244.177	520.826	265.938
1450660_at	cereblon	Crbn	583.019	211.995	807.005	457.922
1426978_at	cullin 1	Cul1	269.197	257.055	353.762	242.957
1445499_at	cullin 2	Cul2	349.093	295.486	86.373	31.705
1426265_x_at	cullin 3	Cul3	1003.425	1031.293	828.663	838.795
1460171_at	cullin 4A	Cul4a	186.388	237.725	146.405	194.176
1424948_x_at	cullin 4B	Cul4b	178.630	265.995	76.875	146.645
1449283_a_at	cullin 5	Cul5	357.934	168.112	208.013	178.684
1424209_at	cullin 7	Cul7	199.551	138.945	198.650	133.838
1423995_at	DET1 and DDB1 associated 1	Dda1	524.836	384.563	614.869	588.697
1451572_a_at	damage specific DNA binding protein 1	Ddb1	351.991	452.426	399.597	427.034
1423811_at	damage specific DNA binding protein 2	Ddb2	59.508	64.962	84.630	50.856
1456256_at	diablo homolog (Drosophila)	Diablo	741.719	338.990	209.305	457.168
1420509_at	deltex 3 homolog (Drosophila)	Dtx3	246.606	213.608	675.840	294.017
1435873_a_at	deltex 4 homolog (Drosophila)	Dtx4	171.318	239.864	337.084	178.162
1455610_at	DAZ interacting protein 3, zinc finger	Dzip3	80.914	67.698	585.631	17.472
1419866_s_at	ectodermal-neural cortex 1	Enc1	328.139	114.140	4653.486	633.412
1434933_at	excision repaiross-complementing rodent repair deficiency, complementation group 8	Ercc8	56.821	43.809	47.399	34.816
1436305_at	Fanconi anemia, complementation group L	Fancl	173.345	162.839	90.224	8.850
1429054_at	F-box and leucine-rich repeat protein 12	Fbxl12	60.988	47.986	49.552	46.648
1439153_at	F-box and leucine-rich repeat protein 14	Fbxl14	89.807	128.902	150.311	122.259
1417446_at	F-box and leucine-rich repeat protein 17	Fbxl17	349.161	270.414	490.315	167.295
1429159_at	F-box and leucine-rich repeat protein 3	Fbxl3	304.722	297.294	7.571	238.665
1434115_at	F-box and leucine-rich repeat protein 4	Fbxl4	85.718	248.676	183.340	184.835
1416278_a_at	F-box and leucine-rich repeat protein 5	Fbxl5	181.707	386.527	450.572	234.368
1418371_at	F-box protein 11	Fbxo11	164.647	267.477	559.730	358.155
1457139_at	F-box protein 18	Fbxo18	403.535	278.625	282.859	466.676
1443466_s_at	F-box protein 21	Fbxo21	193.762	294.061	545.289	584.576
1430987_s_at	F-box protein 22	Fbxo22	674.953	1437.684	605.848	674.711
1435211_at	F-box protein 25	Fbxo25	150.863	360.242	545.002	127.312
1420895_at	F-box protein 3	Fbxo3	383.645	354.483	347.270	358.077
1436982_at	F-box protein 30	Fbxo30	182.692	85.973	100.470	380.661
1435104_at	F-box protein 31	Fbxo31	161.809	55.370	129.441	169.100
**1438672_at**	**F-box protein 32**	**Fbxo32**	**1290.847**	**61.359**	**199.998**	**633.889**
1452710_at	F-box protein 33	Fbxo33	153.539	297.530	730.801	256.502
1429370_a_at	F-box protein 6	Fbxo6	482.814	228.496	157.375	283.357
1433675_at	F-box protein 7	Fbxo7	127.087	385.236	157.730	100.563
1452970_at	f-box protein 9	Fbxo9	604.597	731.228	1228.859	780.457
1453186_at	F-box and WD-40 domain protein 11	Fbxw11	2886.837	4702.137	7206.437	3437.328
1422780_at	F-box and WD-40 domain protein 2	Fbxw2	158.212	134.367	140.465	204.588
1437222_x_at	F-box and WD-40 domain protein 4	Fbxw4	247.818	114.971	154.383	183.379
1436174_at	F-box and WD-40 domain protein 5	Fbxw5	152.365	146.274	140.670	134.464

1435140_at	F-box and WD-40 domain protein 7	Fbxw7	617.473	120.733	1561.142	146.987
1448151_at	F-box and WD-40 domain protein 8	Fbxw8	222.308	263.536	103.950	260.251
1424755_at	feminization 1 homolog b (C. elegans)	Fem1b	283.110	380.834	37.684	279.608
1417605_s_at	glucuronidase, beta	Gusb	131.965	252.513	69.160	213.462
1439421_x_at	HECT domain containing 1	Hectd1	878.940	808.238	819.907	921.977
1416155_at	hect (homologous to the E6-AP (UBE3A) carboxyl terminus) domain and RCC1 (CHC1)-like domain (RLD) 1	Herc1	213.401	122.859	197.048	232.033
1423635_at	hect (homologous to the E6-AP (UBE3A) carboxyl terminus) domain and RCC1 (CHC1)-like domain (RLD) 2	Herc2	208.448	123.200	428.087	239.384
1452358_at	hect domain and RLD 3	Herc3	154.991	192.102	529.416	121.128
1455504_a_at	hect domain and RLD 4	Herc4	349.238	574.517	223.580	240.624
1419757_at	helicase-like transcription factor	Hltf	232.210	1234.311	221.207	147.544
1455255_at	HECT, UBA and WWE domain containing 1	Huwe1	420.336	310.249	423.077	491.455
1415962_at	interleukin-1 receptor-associated kinase 1	Irak1	629.992	699.335	372.786	725.826
**1434170_at**	**kelch repeat and BTB (POZ) domain containing 10**	**Kbtbd10**	**327.819**	**6.628**	**7.125**	**19.776**
1432466_a_at	kelch repeat and BTB (POZ) domain containing 7	Kbtbd7	260.361	480.477	137.323	205.464
1426460_a_at	potassium channel tetramerisation domain containing 10	Kctd10	208.630	136.555	164.034	447.695
1426760_at	potassium channel tetramerisation domain containing 13	Kctd13	63.897	81.972	559.581	70.144
1434034_at	kelch-like ECH-associated protein 1	Keap1	318.735	282.248	337.567	337.894
1436839_at	kelch-like 12 (Drosophila)	Klhl12	267.575	199.955	350.203	135.262
1434225_at	kelch-like 13 (Drosophila)	Klhl13	498.995	117.126	692.424	231.474
1424835_at	kelch-like 2, Mayven (Drosophila)	Klhl2	60.629	334.535	381.860	155.495
1425309_at	kelch-like 20 (Drosophila)	Klhl20	132.512	140.679	169.917	120.237
1429061_at	kelch-like 21 (Drosophila)	Klhl21	309.468	102.215	64.906	112.200
1437067_at	kelch-like 22 (Drosophila)	Klhl22	293.719	487.551	875.896	382.624
**1427921_s_at**	**kelch-like 24 (Drosophila)**	**Klhl24**	**1151.297**	**446.539**	**386.560**	**448.672**
1428913_at	kelch-like 7 (Drosophila)	Klhl7	719.392	441.279	753.932	397.905
1448305_at	kelch-like 9 (Drosophila)	Klhl9	1050.421	1208.184	1434.442	877.012
**1447809_x_at**	**LIM domain only 7**	**Lmo7**	**1624.454**	**647.634**	**233.860**	**225.509**
1456070_at	ligand of numb-protein X 1	Lnx1	357.865	282.544	521.633	313.516
1427039_at	leucine rich repeat containing 41	Lrrc41	124.316	149.533	168.929	151.902
1422516_a_at	leucine rich repeat and sterile alpha motif containing 1	Lrsam1	73.962	71.729	217.401	74.842
1416180_a_at	mitogen-activated protein kinase kinase kinase 1	Map3k1	99.563	97.239	48.906	15.656
1423431_a_at	transformed mouse 3T3 cell double minute 2	Mdm2	292.696	291.931	13.513	19.979
1454758_a_at	transformed mouse 3T3 cell double minute 4	Mdm4	61.159	45.552	17.134	19.732
1451469_at	mex3 homolog C (C. elegans)	Mex3c	292.787	432.994	542.843	466.786

1415881_at	mahogunin, ring finger 1	Mgrn1	494.719	296.093	384.935	272.633
1451983_at	mindbomb homolog 1 (Drosophila)	Mib1	211.143	377.914	792.348	319.363
1417266_at	mindbomb homolog 2 (Drosophila)	Mib2	121.951	108.713	212.237	153.742
1427212_at	makorin, ring finger protein, 1	Mkrn1	399.006	426.087	553.792	162.748
1415830_at	makorin, ring finger protein, 2	Mkrn2	514.301	271.114	372.654	288.140
1423642_at	menage a trois 1	Mnat1	227.916	193.440	259.993	149.647
1452047_at	male-specific lethal 2 homolog (Drosophila)	Msl2	426.612	368.932	542.275	427.610
1429707_at	mitochondrial ubiquitin ligase activator of NFKB 1	Mul1	219.511	184.305	174.487	250.457
1458458_at	MYC binding protein 2	Mycbp2	687.071	664.780	1024.328	701.936
1420711_a_at	myosin regulatory light chain interacting protein	Mylip	627.678	1232.135	465.260	757.360
1449214_a_at	neural precursor cell expressed, developmentally down-regulated 4	Nedd4	2286.678	1695.245	2507.258	2657.081
1452078_a_at	neural precursor cell expressed, developmentally down-regulated gene 4-like	Nedd4l	147.495	330.726	1053.426	30.136
1436883_at	nuclear transcription factor, X-box binding 1	Nfx1	117.985	567.500	86.387	167.756
1452189_at	NHL repeat containing 1	Nhlrc1	67.433	40.288	165.864	42.544
1452331_s_at	nitric oxide synthase interacting protein	Nosip	142.106	133.603	209.403	138.801
1418262_at	osteopetrosis associated transmembrane protein 1	Ostm1	333.930	321.122	634.928	240.215
1416229_at	polycomb group ring finger 3	Pcgf3	403.665	300.763	338.134	260.180
1430271_x_at	PDZ domain containing RING finger 3	Pdzrn3	528.525	356.997	257.673	516.227
1423248_at	pellino 1	Peli1	304.077	244.370	6.531	6.605
1451784_x_at	pellino 2	Peli2	88.150	253.663	44.983	76.478
1416526_a_at	peroxisomal biogenesis factor 12	Pex12	79.726	167.529	103.228	92.759
1427888_a_at	pleckstrin homology domain interacting protein	Phip	281.857	222.411	39.516	168.098
1435517_x_at	protein inhibitor of activated STAT 1	Pias1	249.878	270.913	112.793	104.712
1429302_at	protein inhibitor of activated STAT 2	Pias2	144.790	228.077	78.894	151.007
1428919_at	protein inhibitor of activated STAT 4	Pias4	146.972	158.391	152.704	154.142
1428926_at	praja1, RING-H2 motif containing	Pja1	142.209	295.179	245.775	89.396
1434703_at	praja 2, RING-H2 motif containing	Pja2	1150.193	628.277	3424.935	757.731
1417453_at	peroxisomal membrane protein 3	Pxmp3	413.096	593.637	266.694	563.596
1456846_at	RAN binding protein 2	Ranbp2	431.862	358.251	418.152	386.086
1434134_at	RanBP-type and C3HC4-type zinc finger containing 1	Rbck1	194.064	151.143	174.964	240.980
1418524_at	ring-box 1	Rbx1	3003.428	2282.119	2201.178	2837.173
1424416_at	RING CCCH (C3H) domains 1	Rc3h1	225.252	155.544	265.663	200.899
1451504_at	ring finger and CCCH-type zinc finger domains 2	Rc3h2	382.979	341.249	717.304	61.510
**1439772_at**	**ring finger and WD repeat domain 2**	**Rfwd2**	**1204.010**	**656.407**	**702.478**	**820.378**
1415780_a_at	Rho-related BTB domain containing 1	Rhobtb1	376.585	216.908	30.947	282.165
1448526_at	Rho-related BTB domain containing 2	Rhobtb2	149.263	125.517	171.329	107.195
1435742_at	Rho-related BTB domain containing 3	Rhobtb3	158.047	66.876	317.323	105.127
**1431255_at**	**ring finger protein 10**	**Rnf10**	**1322.710**	**673.291**	**599.855**	**812.591**

1435730_at	ring finger protein 103	Rnf103	284.633	562.981	440.195	305.140
1454680_at	ring finger protein 11	Rnf11	1486.251	2010.531	2178.354	1573.979
1460401_at	ring finger 111	Rnf111	168.444	252.158	301.568	206.320
1450894_a_at	ring finger protein 113A2	Rnf113a2	106.023	148.835	127.726	83.108
1437405_a_at	ring finger protein 114	Rnf114	839.610	663.737	562.192	400.603
1416252_at	ring finger protein 115	Rnf115	358.111	602.933	844.237	481.347
1456054_a_at	ring finger protein 121	Rnf121	103.315	118.483	180.536	77.056
1435358_at	ring finger protein 123	Rnf123	366.883	224.906	332.959	243.608
1434954_at	ring finger protein 125	Rnf125	169.778	68.538	61.341	299.462
**1435335_a_at**	**ring finger protein 128**	**Rnf128**	**1561.058**	**1998.183**	**352.785**	**107.207**
1424048_a_at	ring finger protein 13	Rnf13	648.004	1103.799	805.562	474.252
1455807_at	ring finger protein 130	Rnf130	514.555	831.932	466.728	489.955
1433570_s_at	ring finger protein 139	Rnf139	810.111	594.406	464.511	565.791
1416220_at	ring finger protein 14	Rnf14	599.732	591.495	1759.845	434.574
1415757_at	ring finger protein 144A	Rnf144a	334.393	188.472	231.614	161.658
1438659_x_at	ring finger protein 144B	Rnf144b	229.880	59.983	14.389	161.658
1440346_at	ring finger protein 145	Rnf145	184.416	735.755	976.259	370.171
1441814_s_at	ring finger protein 146	Rnf146	961.918	507.088	1032.658	648.204
1426696_at	ring finger protein 166	Rnf166	567.120	428.833	374.159	472.076
1455476_a_at	ring finger protein 167	Rnf167	289.128	348.750	363.451	349.122
1454807_a_at	ring finger protein 168	Rnf168	288.345	210.703	424.313	190.879
1454677_at	ring finger protein 170	Rnf170	147.745	114.987	260.479	111.483
1416230_at	ring finger protein 181	Rnf181	764.109	3419.184	1018.044	709.631
1449362_a_at	ring finger protein 187	Rnf187	1167.746	1467.228	2874.984	1441.631
1438289_a_at	ring finger protein 19A	Rnf19a	163.606	226.672	29.873	25.421
1424203_at	ring finger protein 19B	Rnf19b	126.454	120.692	121.136	32.549
1416520_x_at	ring finger protein 2	Rnf2	155.346	208.064	134.245	90.352
1457351_at	ring finger protein 20	Rnf20	153.222	230.238	31.982	30.476
**1459800_s_at**	**ring finger protein 207**	**Rnf207**	**255.064**	**33.652**	**26.676**	**31.535**
1448961_at	ring finger protein 219	Rnf219	81.504	75.377	7.464	5.953
1435488_at	ring finger protein 220	Rnf220	129.836	129.362	292.300	125.594
1456736_x_at	ring finger protein 31	Rnf31	161.455	117.956	111.799	102.134
1450626_at	ring finger protein 34	Rnf34	461.269	314.350	619.816	261.904
1449408_at	ring finger protein 38	Rnf38	99.209	117.595	12.841	103.490
1419181_at	ring finger protein 4	Rnf4	115.967	221.360	109.457	168.979
1450655_at	ring finger protein 40	Rnf40	243.587	237.417	232.560	250.062
1415988_at	ring finger protein 44	Rnf44	352.938	500.103	604.512	495.432
1433432_x_at	ring finger protein 5	Rnf5	444.260	800.709	439.085	503.926
1440886_at	ring finger protein (C3H2C3 type) 6	Rnf6	540.269	639.627	647.122	416.306
1455724_at	ring finger protein 7	Rnf7	2648.474	2145.998	2342.841	2270.391
1455928_x_at	ring finger and SPRY domain containing 1	Rspry1	93.081	308.588	176.542	148.552
1441972_at	Seven in absentia 1A (Siah1a), mRNA	Siah1a	327.003	216.712	35.959	182.893
1437629_at	seven in absentia 2	Siah2	122.869	204.267	234.722	62.262
**1436015_s_at**	**S-phase kinase-associated protein 1A**	**Skp1a**	**3114.886**	**5034.868**	**4772.805**	**2679.420**

1416846_a_at	SMAD specific E3 ubiquitin protein ligase 1	Smurf1	128.006	77.830	89.308	111.320
1417612_at	SMAD specific E3 ubiquitin protein ligase 2	Smurf2	297.671	439.053	89.308	347.941
**1438504_x_at**	**suppressor of cytokine signaling 2**	**Socs2**	**825.745**	**325.219**	**161.346**	**244.125**
1426276_at	suppressor of cytokine signaling 3	Socs3	155.001	51.041	34.835	142.987
1424235_at	suppressor of cytokine signaling 4	Socs4	203.478	406.205	237.513	328.673
1420634_a_at	suppressor of cytokine signaling 5	Socs5	162.788	264.254	455.978	210.961
1448248_at	suppressor of cytokine signaling 6	Socs6	78.591	110.528	100.567	65.028
1418330_at	speckle-type POZ protein	Spop	2137.066	1333.921	2887.037	1770.010
1418144_a_at	SPRY domain-containing SOCS box protein SSB-1	Spsb1	327.151	74.365	182.863	516.148
1424114_s_at	splA/ryanodine receptor domain and SOCS box containing 4	Spsb4	183.185	625.223	99.568	44.696
1435484_at	STIP1 homology and U-Box containing protein 1	Stub1	551.754	918.600	1784.240	1021.728
1452769_at	synovial apoptosis inhibitor 1, synoviolin	Syvn1	146.630	229.368	295.636	159.532
1427901_at	transcription elongation factor B (SIII), polypeptide 1	Tceb1	137.710	97.356	497.914	103.728
**1416367_at**	**transcription elongation factor B (SIII), polypeptide 2**	**Tceb2**	**3386.650**	**2534.605**	**2911.537**	**2569.120**
1433824_x_at	tumor necrosis factor, alpha-induced protein 1 (endothelial)	Tnfaip1	639.067	617.349	436.866	685.400
1456205_x_at	topoisomerase I binding, arginine/serine-rich	Topors	269.760	568.455	398.309	365.738
1448159_at	TNF receptor-associated factor 3	Traf3	230.395	201.700	535.383	187.928
1452540_a_at	TNF receptor-associated factor 6	Traf6	79.209	100.804	80.734	83.948
1424199_at	tripartite motif-containing 11	Trim11	147.207	135.520	144.224	128.942
1434743_x_at	tripartite motif-containing 13	Trim13	152.790	160.534	121.359	71.673
1451356_at	tripartite motif-containing 2	Trim2	72.291	849.286	1530.150	225.319
1451093_at	tripartite motif-containing 21	Trim21	249.861	186.104	72.018	137.859
1455310_at	tripartite motif-containing 23	Trim23	116.025	150.622	265.405	84.457
1460363_at	tripartite motif-containing 24	Trim24	103.589	147.451	114.283	140.081
1424748_at	tripartite motif-containing 25	Trim25	562.281	1052.276	138.578	139.309
1436868_at	tripartite motif-containing 27	Trim27	447.268	691.598	594.826	294.703
1448555_at	tripartite motif-containing 28	Trim28	920.217	901.883	1162.815	1574.574
1439897_at	tripartite motif-containing 3	Trim3	197.615	177.610	857.474	260.135
1424597_at	tripartite motif-containing 32	Trim32	125.542	216.581	1489.481	152.748
1427929_a_at	tripartite motif-containing 33	Trim33	224.853	369.662	385.372	294.519
1422603_at	tripartite motif-containing 37	Trim37	427.518	304.312	591.347	225.795
1428151_x_at	tripartite motif-containing 39	Trim39	126.342	94.668	158.412	113.352
1446090_at	tripartite motif-containing 44	Trim44	437.035	842.366	1987.398	718.962
**1416238_at**	**tripartite motif-containing 54**	**Trim54**	**1615.012**	**37.992**	**42.144**	**92.062**
**1423216_a_at**	**tripartite motif-containing 63**	**Trim63**	**1524.068**	**344.437**	**12.950**	**53.950**
1423177_a_at	tripartite motif-containing 68	Trim68	115.566	41.078	38.255	59.733
1440338_at	tripartite motif protein 8	Trim8	108.600	173.286	255.749	111.278
1452032_at	thyroid hormone receptor interactor 12	Trip12	599.265	543.115	327.871	483.195

1418162_at	tetraspanin 17	Tspan17	50.926	54.992	24.430	67.262
1422554_at	tetratricopeptide repeat domain 3	Ttc3	947.626	434.508	6984.575	509.509
1460561_x_at	ubiquitin C	Ubc	6958.155	8403.687	7017.093	7025.037
1415671_at	ubiquitin protein ligase E3A	Ube3a	921.097	627.695	1097.388	637.158
1454990_at	ubiquitin protein ligase E3B	Ube3b	257.988	219.530	209.882	232.591
1436703_x_at	ubiquitin protein ligase E3C	Ube3c	194.557	136.553	217.931	144.285
1416563_at	ubiquitination factor E4A, UFD2 homolog (S. cerevisiae)	Ube4a	101.451	113.965	93.910	140.356
1417002_at	ubiquitination factor E4B, UFD2 homolog (S. cerevisiae)	Ube4b	288.628	354.675	112.209	110.991
1417718_at	ubiquitin protein ligase E3 component n-recognin 1	Ubr1	252.909	244.821	433.815	248.945
1439375_x_at	ubiquitin protein ligase E3 component n-recognin 2	Ubr2	537.318	237.324	326.764	298.356
1418225_at	ubiquitin protein ligase E3 component n-recognin 3	Ubr3	626.386	662.967	725.973	647.978
1416381_a_at	ubiquitin protein ligase E3 component n-recognin 5	Ubr5	888.206	748.914	813.983	985.355
1416274_at	ubiquitin protein ligase E3 component n-recognin 7 (putative)	Ubr7	236.825	175.420	222.588	140.735
1424524_at	ubiquitin-like, containing PHD and RING finger domains 2	Uhrf2	109.743	139.619	145.790	120.750
1456551_at	unkempt homolog (Drosophila)	Unk	63.192	66.837	68.204	67.752
1427886_at	unkempt-like (Drosophila)	Unkl	102.553	355.804	394.044	97.665
1423144_at	Vpr (HIV-1) binding protein	Vprbp	264.912	205.602	226.290	204.362
1419062_at	vacuolar protein sorting 11 (yeast)	Vps11	111.072	139.029	186.138	134.986
1448270_at	vacuolar protein sorting 41 (yeast)	Vps41	326.769	487.225	244.487	393.400
1423517_at	vacuolar protein sorting 8 homolog (S. cerevisiae)	Vps8	205.694	352.918	124.360	149.228
1421855_at	Wolf-Hirschhorn syndrome candidate 1 (human)	Whsc1	55.303	77.989	100.221	62.745
1450849_at	WD repeat and SOCS box-containing 1	Wsb1	61.427	146.924	86.087	100.402
1431416_a_at	WW domain containing E3 ubiquitin protein ligase 1	Wwp1	548.490	1392.450	178.926	369.667
1442027_at	X-linked inhibitor of apoptosis	Xiap	692.748	739.100	77.007	20.769
**1455914_at**	**zinc finger and BTB domain containing 16**	**Zbtb16**	**619.993**	**70.926**	**200.665**	**227.719**
1417383_at	zinc and ring finger 1	Znrf1	168.722	89.146	258.765	155.011
1460685_at	zinc and ring finger 2	Znrf2	228.133	984.565	191.578	245.232
1422677_at	zyg-ll homolog B (C. elegans)	Zyg11b	623.125	413.132	339.629	441.357

Data are expressed as mean+standard deviation (SD not shown). Heart: n=3; kidney: n=2; Brain: n=2; Aorta: n=3
